# A prospective randomized open-label crossover trial of regional citrate anticoagulation vs. anticoagulation free liver dialysis by the Molecular Adsorbents Recirculating System

**DOI:** 10.1186/cc11180

**Published:** 2012-02-03

**Authors:** Björn Meijers, Wim Laleman, Pieter Vermeersch, Frederik Nevens, Alexander Wilmer, Pieter Evenepoel

**Affiliations:** 1 Department of Internal Medicine, Nephrology, University Hospitals Leuven, Herestraat 49, Leuven, B-3000, Belgium; 2 Department of Internal Medicine, Hepatology, University Hospitals Leuven, Herestraat 49, Leuven, B-3000, Belgium; 3 Laboratory Medicine, Immunology, University Hospitals Leuven, Herestraat 49, Leuven, B-3000, Belgium; 4 Department of Internal Medicine, medical intensive care unit, University Hospitals Leuven, Herestraat 49, Leuven, B-3000, Belgium

## Abstract

**Introduction:**

The Molecular Adsorbent Recycling System (MARS) is used to treat patients with liver failure. Observational data suggest that citrate anticoagulation during MARS is feasible. Comparative studies on the optimal anticoagulation regimen during MARS are lacking. The aim of the current study was to evaluate two heparin-free anticoagulation regimens.

**Methods:**

We performed a prospective randomized open-label crossover study of regional citrate anticoagulation against no anticoagulation. Ten patients (age 55 ± 11 years) with liver failure undergoing MARS treatment were included. The primary endpoint was completion of MARS sessions. Secondary endpoints included treatment efficacy and safety. Longevity of MARS treatment was plotted as a Kaplan-Meier estimate. Fisher's exact test was used for contingency table analysis.

**Results:**

Of a total of 27 6-hour sessions, four sessions had to be terminated prematurely, three due to occlusive clotting of the extracorporeal circuit and one due to uncontrollable bleeding from the vascular access site. All four events occurred in the group without anticoagulation. Between group comparison demonstrated citrate anticoagulation to significantly increase the likelihood of completed MARS treatment (Fisher's exact test, *P *0.04). This translates into higher bilirubin reduction ratios when citrate was applied (reduction ratio 0.25 vs. 0.15, *P *0.02). Systemic ionized calcium concentrations were significantly reduced during citrate anticoagulation (*P *< 0.001) but remained within a safe range. We observed no major adverse events.

**Conclusions:**

Regional citrate anticoagulation in patients with liver failure is feasible. Citrate anticoagulation provides superior patency of the extracorporeal circuit. Avoidance of anticoagulation during MARS results in significant loss of treatment efficacy, due to treatment downtime. Additional studies are required to identify the optimal anticoagulation regimen for extracorporeal circulation in patients with liver failure.

## Introduction

Liver failure, whether acute, acute-on-chronic or end-stage liver insufficiency, remains an important cause of morbidity and mortality. The lack of liver detoxification as well as the loss of metabolic and regulatory functions of the liver leads to life-threatening complications, including renal failure, altered immune response, hepatic coma and systemic hemodynamic dysfunction, eventually culminating in multi-organ failure. Current medical therapy involves the management of the precipitating event and treatment of complications until the liver eventually recovers, leaving us with no other treatment options other than transplantation if these attempts fail [1].

During the acute phase of their disease, a substantial number of patients are treated using extracorporeal blood purification devices. Besides regular hemodialysis, the Molecular Adsorbent Recycling System (MARS, Gambro AB, Lund, Sweden) is used with increasing frequency. This non-biologic liver dialysis system is currently in use to bridge patients with acute liver failure and acute-on-chronic liver failure, either to recovery of native liver function or to liver transplantation [2].

Liver failure is characterized by a balanced but brittle coagulation system. Minor disturbances profoundly increase the risks of severe bleeding and of thrombotic events [3]. An important area of debate at present is the choice of the optimal extracorporeal blood circuit anticoagulation regimen. On the one hand, systemic anticoagulation therapy should be minimized given the high risk of bleeding associated with liver failure. On the other hand, contact of blood with the extracorporeal circuit leads to coagulation activation and might result in loss of coagulation factors, contributing to broad coagulation disturbances [4].

Unfractionated heparin and to a lesser extent low molecular weight heparin are the standard of care anticoagulation regimens in most centers. However, optimal dosage has not been studied. Moreover, heparin induces systemic anticoagulation, thereby aggravating liver failure-associated coagulopathy. Several retrospective studies reported on protocols to minimize heparin anticoagulation during MARS dialysis. Faybik *et al*. reported on the use of prostaglandin I_2 _during MARS treatment. However, in this cohort, half of the patients still received additional unfractionated heparin [5]. Several retrospective studies suggested the feasibility of MARS without anticoagulation [6-10]. All of these studies reported clotting of the extracorporeal circuit and/or severe coagulopathy due to contact activation in a subset of treatments (Table 1).

**Table 1:** Heparin-free MARS studies

Study	Design	Indication	Total n	Heparin Free patients/sessions	Clotting n (P/S %)	Coagulopathy or Bleeding n (P/S %)	References
Doria et al. (2004)	retrospective	AoCLF/pruritus	9	9/74	0 (0/0%)	4 (44/nd%)	[6]
Tan et al. (2007)^a^	retrospective	ALF/AoCLF	4	4/12	1 (25/8%)	1 (25/8%)	[8]
Bachli et al. (2007)	retrospective	ALF/AoCLF/pruritus	21	nd/40	nd	7 (nd/18%)	[7]
Yang et al (2008)^a^	retrospective	ALF/AoCLF	10	5/13	1 (20/8%)	1 (20/8%)	[9]
Tan et al. (2010)^a^	retrospective	ALF/AoCLF	12	12/44	11 (nd/25%)	4 (nd/9%)	[10]
Faybik et al.(2011)	Prospective	ALF/AoCLF	20	20/77	1 (5/1%)	1 (5/1%)	[15]

^a^These papers originate from the same research group. It is unclear whether these manuscripts report on the same patient population or not. ALF, acute liver failure; AoCLF, acute-on-chronic liver failure; MARS, Molecular Adsorbents Recirculation System; P, patients; S, sessions; nd, no data.

Regional citrate anticoagulation might provide sufficient anticoagulation of the extracorporeal blood circuit, thus minimizing contact activation-associated coagulopathy, while maintaining systemic coagulation [11,12]. Regional citrate anticoagulation for regular hemodialysis is feasible, well-tolerated and safe [11]. In contrast, experience with citrate anticoagulation during treatment of patients with liver failure is limited. The liver contributes substantially to exogenous citrate metabolism and endogenous citrate clearance in patients with liver failure is reduced [13]. In an observational non-comparative study, regional citrate anticoagulation was used during 50 consecutive liver dialysis treatments using the fractionated plasma separation and adsorption system (FPSA, Prometheus^®^). Substantial clotting of the dialyser occurred only twice [14]. More recently, Faybik *et al*. performed a prospective observational study in twenty patients treated with MARS. Regional citrate anticoagulation appeared safe with superior patency of the extracorporeal circuit during treatment [15], but further studies are required to support widespread application of citrate anticoagulation during MARS treatment [16].

Little evidence thus supports choice of the optimal anticoagulation regimen during MARS. The aims of this prospective cross-over study in patients with acute-on-chronic liver failure were (A) to investigate feasibility of regional citrate anticoagulation during MARS and (B) to compare biocompatibility, safety and efficacy of anticoagulation-free versus regional citrate anticoagulation for anticoagulation during MARS.

## Materials and methods

### Patients

Patients with acute-on-chronic liver failure, admitted to the medical intensive care unit, University Hospitals Leuven, Belgium, were screened for eligibility. The diagnosis of liver failure was considered if patients, previously known with compensated chronic liver disease as defined by Gines *et al*. [17], developed intrahepatic cholestasis (serum bilirubin > 5 mg/dl without radiologic evidence of extra-hepatic origin) and had at least one of the following complications within a time span of four to eight weeks after a potential identifiable acute superposed hepatic insult: a) a progressive hyperbilirubinemia defined as a more than 50% increase of bilirubin or up to a level of > 20 mg/dl, b) hepatic encephalopathy ≥ II, c) *de novo *development of ascites, and/or d) hepatorenal syndrome, defined according to the criteria of the International Ascites Club which were prevalent at that time [18]. Inclusion criteria were scheduled liver dialysis using the MARS system and age over 18 years. Exclusion criteria were severe hypocalcemia (Ca^2+ ^< 0.9 mmol·l^-1^) or acidosis (pH < 7.25) due to any cause.

### Study design

This was a prospective randomized open-label double crossover study (clinicaltrials.gov NCT00695617). The cross-over design was chosen to minimize heterogeneity, given that liver failure is a heterogeneous disease state and given the low number of patients available for enrolment.

At day 1, patients were randomized to either MARS treatment with citrate anticoagulation (*n *= 5) or coagulation-free MARS treatment (*n *= 5). At day 2, patients were switched to the opposite anticoagulant regimen. From day 3 and thereafter, patients received their initial anticoagulation regimen. Randomization was by the sealed envelope method. Written informed consent was obtained from all patients, or in case of hepatic encephalopathy, from their next of kin. The study was performed according to the Declaration of Helsinki and approved by the ethics review board of the University Hospital Leuven.

### Procedures

**Figure 1:**
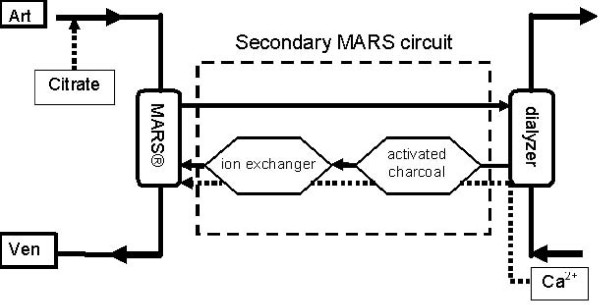
**Schematic diagram of the Molecular Adsorbents Recirculation System**. Blood is dialysed against an albumin containing secondary circuit. The MARS^® ^membrane allows passage of both water-soluble and the free fraction of protein-bound hepatic failure related toxins. In the secondary circuit, the albumin solution is regenerated by low-flux dialysis and passage over two adsorbent surfaces, an activated charcoal and an anion exchanger, respectively. For regional citrate anticoagulation, citrate is continuously infused in the afferent blood tubing, thus reducing ionized calcium concentrations at the MARS dialyzer inlet to 0.2 to 0.3 mmol·l^-1^. Restoration of blood calcium is by dialysis against dialysate containing Ca^2+ ^at a concentration of 1.5 mmol·l^-1^. Systemic ionized calcium concentrations are repeatedly monitored during the procedure. If systemic calcium concentration falls below 0.8 mmol·l^-1 ^despite calcium influx through the dialysate, additional CaCl_2 _is administered as an intravenous bolus injection. MARS, Molecular Adsorbents Recirculation System.

During MARS treatment, both water-soluble and the free fraction of protein-bound liver toxins are dialysed into the secondary circuit (Figure 1), while blood cells and proteins are retained in the primary circuit. In the secondary circuit, an albumin-rich aqueous solution (human albumin 200 g/l, Baxter SA, Lessines, Belgium) is regenerated over two adsorbent columns. A dialyser in the secondary circuit permits clearance of water-soluble molecules [2,19,20]. MARS treatments were delivered with a MARS monitor (Gambro AB, Lund, Sweden) coupled to a hemodialysis device (AK200, Gambro AB, Lund, Sweden). MARS prescription was six consecutive hours for all procedures. The blood circuit was primed with 5000 IE heparin (LEO pharma, Wilrijk, Belgium). Blood access was by double lumen catheterization (Hemoaccess, Gambro-Hospal, Zaventem, Belgium) of the femoral vein in all patients.

Venous blood samples for monitoring of pH and ionized calcium concentrations were taken from the dialysis tubing at sampling ports proximal and distal to citrate infusion at start, 30, 60, 120, 240 and 360 (or end of treatment) minutes. Samples were collected in dedicated recipients (PICO50, Radiometer Copenhagen, Brønshøj, Denmark, containing 80 IU of electrolyte-balanced heparin) and immediately analyzed on an ABL 700 blood gas analyzer (Radiometer Copenhagen, Brønshøj, Denmark). For validation purposes, simultaneous collection of venous and arterial blood samples was performed in a subset of patients.

For citrate anticoagulation, hypertonic trisodium citrate (1.035 M, Baxter, Lessines, Belgium) was continuously infused into the afferent dialysis tubing. Citrate flow rates, initially set at 62.1 mmol·h^-1^, were adapted to maintain extracorporeal ionized calcium concentrations measured at the dialyzer inlet between 0.20 and 0.30 mmol·l^-1^. The anticoagulant effect was neutralized by dialyzing against a calcium-containing dialysate. Dialysate composition was Na^+ ^135 mmol·l^-1^, K^+ ^3 mmol·l^-1^, Ca^2+ ^1.5 mmol·l^-1^, Mg^2+ ^0.5 mmol·l^-1^, Cl^- ^108 mmol·l^-1^, HCO_3_^- ^25 mmol·l^-1 ^and acetate 3 mmol·l^-1^. Systemic ionized calcium concentrations and blood pH, sampled from the afferent dialysis tubing proximal to citrate infusion, were monitored at the start and at 30, 60,120, 240 and 360 minutes. Systemic ionized calcium concentrations were maintained within ± 15% of baseline values. According to a prespecified protocol, CaCl_2 _(10 mL, 11 mEq Ca^2+^, Sandoz, Lausanne, Switzerand) was administered as an intravenous bolus injection when ionized calcium concentrations dropped below 0.8 mmol·l^-1^.

During sessions without anticoagulation, dialysate composition was Na^+ ^140 mmol·l^-1^, K^+ ^3 mmol·l^-1^, Ca^2+ ^1.5 mmol·l^-1^, Mg^2+ ^0.5 mmol·l^-1^, Cl^- ^108 mmol·l^-1^, HCO_3_^- ^38 mmol·l^-1 ^and acetate 3 mmol·l^-1^.

### Outcomes

The primary endpoint was uninterrupted completion of MARS sessions. Causes of preterm interruption of MARS treatment, including occlusive clotting of the extracorporeal circuit and intractable bleeding during MARS, were prospectively recorded.

Secondary endpoints included a semi-quantitative dialyser clotting score, which has been used previously [11]. Coagulation studies included activated partial thromboplastin time (aPTT), prothrombin time (PT) measured by standard laboratory techniques and thrombin-antithrombin complex (TAT) formation, analyzed by Enzygnost TAT micro kit (OWMG 15 Dade Behring, Marburg, Germany). Treatment efficacy was defined as reduction ratios (RR) of urea, creatinine, total bilirubin and bile acids. RRs were calculated as RR *= *(*C*_pre_-*C_post_*)/*C*_pre _, where *C*_pre _and *C*_post _represent concentrations at the start and at the end of the treatment session, respectively.

### Statistics

Continuous variables are expressed as mean (standard deviation (SD)) for normally distributed variables or median (interquartile range), otherwise. Due to a wide variation in the prevalence of reported clotting and bleeding episodes (Table 1) we did not perform formal power calculations. The number of patients, but not of sessions, was pre-specified (*n *= 10). Longevity of MARS treatment was plotted as a Kaplan-Meier estimate. Due to low event numbers, Fisher's exact test was used for contingency table analysis. For between group comparison of continuous variables, the Mann-Whitney-Wilcoxon test was used. A two sided *P *< 0.05 was considered significant. For statistics, SAS (version 9.1, the SAS institute, Cary, NC, USA) and SPSS (version 15.0, Chicago, Il, USA) software packages were used.

## Results

### Study population

Between August 2008 and June 2009, ten patients with acute-on-chronic liver failure, fulfilling the inclusion criteria and providing informed consent, were included in this prospective cross-over study. Baseline characteristics and three-month patient survival are presented in Table 2. Ten patients were randomized to start with citrate anticoagulation (*n *= 5) or to start with heparin-free MARS treatment (*n *= 5). Eight patients received at least one session with both anticoagulation regimens. Two patients died between their first and second treatment session, one treated with citrate anticoagulation, one treated without anticoagulation. As a result, 14 six-hour MARS sessions with regional citrate anticoagulation were compared to 13 six-hour MARS sessions without anticoagulation. The study included eight switches from citrate to anticoagulation-free MARS treatment and seven opposite switches.

**Table 2:** Patient characteristics and 3-month survival

Patient	Age	Etiology	Child	MELD	Day 1	Sessions	3m survival
1	52	Alcoholic	B9	22	citrate	4	deceased
2	47	Alcoholic	C13	43	nil	3	deceased
3	32	Alcoholic	C11	26	nil	3	transplanted, alive
4	68	Hepatitis C	C11	34	citrate	2	lost to follow up
5	51	Alcoholic	C10	23	nil	3	alive
6	63	Alcoholic	C10	30	citrate	4	alive
7	65	Graft failure	B9	23	citrate	3	alive
8	50	NASH	C12	36	nil	3	transplanted, alive
9	64	Alcoholic	C13	34	nil	1	deceased
10	54	α1-AT deficiency	C13	50	citrate	1	deceased

α1-AT deficiency, α1-antitrypsin deficiency; MELD, model for end-stage liver disease

### Primary endpoint analysis

**Figure 2:**
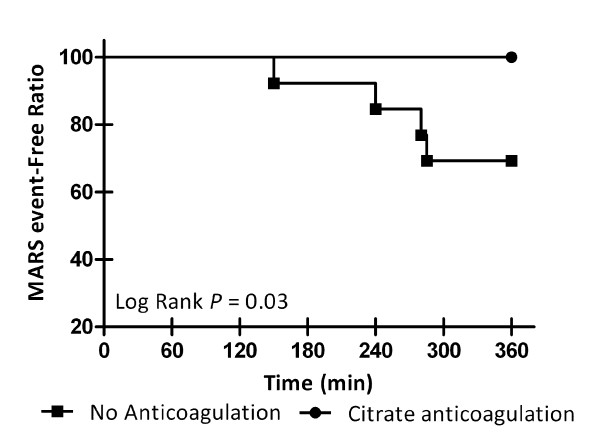
**Kaplan Meier estimate of events during MARS**. Patients were grouped according to anticoagulation regimen during MARS treatment. None of the patients receiving citrate anticoagulation developed an event necessitating preterm cessation of MARS treatment. Four sessions without anticoagulation were interrupted preterm, one due to uncontrollable bleeding and three due to occlusive clotting of the extracorporeal blood circuit. Log Rank *P *= 0.03. MARS, Molecular Adsorbents Recirculation System.

The primary endpoint was completion of scheduled six-hour MARS sessions (*n *= 27). Regional citrate anticoagulation was performed in 14 sessions; the remainder were anticoagulation-free MARS sessions. Four sessions were interrupted preterm, in three patients. During three sessions, occlusive clotting of the extracorporeal circuit occurred. One session was terminated preterm due to uncontrollable bleeding from the vascular access site. All events happened during anticoagulation-free MARS treatments. Longevity of scheduled MARS sessions was plotted as a Kaplan-Meier estimate (Figure 2). Citrate anticoagulation provided significantly fewer interruptions (Log rank, *P *= 0.03). Between group comparison demonstrated citrate anticoagulation significantly increased the likelihood of a full six-hour session of MARS treatment (Fisher's exact test, *P *= 0.04).

### Secondary endpoint analyses

Secondary endpoint analyses are reported in Table 3. Clotting scores indicate more outspoken clotting of the dialyser in sessions without citrate (*P *= 0.009), in line with the primary endpoint of more frequently interrupted MARS sessions without anticoagulation. Although platelets were reduced after most sessions, loss of platelets was more profound after MARS sessions without citrate (*P *= 0.03). Treatment efficacy, defined as RRs, was higher during regional citrate anticoagulation (Table 3). Total bilirubin RR with regional citrate anticoagulation (RR 0.25, SD 0.09) were significantly (*P *= 0.02) higher than without anticoagulation (RR 0.15, SD 0.10). A similar trend was seen for RRs of urea (*P *= 0.06) and creatinine (*P = *0.10). These differences were lost when looking at completed treatment sessions only.

**Table 3:** Comparison of regional citrate anticoagulation versus anticoagulation-free MARS

	Citrate	No anticoagulation	*P*
**Primary endpoint**			
Completed sessions	14/14	9/13	0.04
**Secondary endpoints**			
Clotting score dialyzer (n)			0.009
Clean, white	2	2	
White, limited fibrin deposits, head of dialyzer	7	3	
White, fibrin deposits head and body dialyzer	5	5	
Completely clotted, rinse back successful	0	1	
Completely clotted, rinse back unsuccessful	0	2	
Platelets (x 10^3^/mm^3^)			
Start of treatment	112 (58 - 146)	92 (67 - 169)	0.6
End of treatment	85 (67 - 162)	89 (60 - 117)	0.7
Reduction of platelets (*P *intra-group)	12 (*P *0.08)	28 (*P *0.0005)	0.03
aPTT (s)			
Start of treatment	44.1 (40.5 - 57.2)	57.8 (40.7 - 75.4)	0.5
End of treatment	48.0 (41.3 - 61.5)	52.5 (40.0 - 65.7)	0.6
PT (s)			
Start of treatment	14.2 (13.8 - 16.0)	17.2 (14.6 - 22.0)	0.1
End of treatment	14.5 (14.0 - 16.2)	16.1 (13.8 - 18.3)	0.4
TAT complex (μg/L)			
End of treatment	9.9 (7.7 - 91.7)	25.7 (19.5 - 40.5)	0.3
Increase during treatment	6.1 (5.1 - 50.1)	25.1 (16.8 - 40.5)	0.09
Treatment efficacy, all sessions			
Urea reduction ratio	0.51 (0.14)	0.40 (0.14)	0.06
Creatinine reduction ratio	0.40 (0.13)	0.31 (0.17)	0.10
Bilirubin (total) reduction ratio	0.25 (0.09)	0.15 (0.10)	0.02
Bile acids reduction ratio	0.47 (0.20)	0.42 (0.16)	0.34
Treatment efficacy, completed sessions			
Urea reduction ratio	0.51 (0.14)	0.46 (0.12)	0.4
Creatinine reduction ratio	0.40 (0.13)	0.40 (0.14)	0.9
Bilirubin (total) reduction ratio	0.25 (0.09)	0.18 (0.09)	0.1
Bile acids reduction ratio	0.47 (0.20)	0.46 (0.21)	0.9

aPTT (S), activated partial thromboplastin time (S); MARS, Molecular Adsorbents Recirculation System; PT, prothrombin time (S); TAT complex, thrombin antithrombin complex.

### Feasibility of citrate anticoagulation

**Figure 3:**
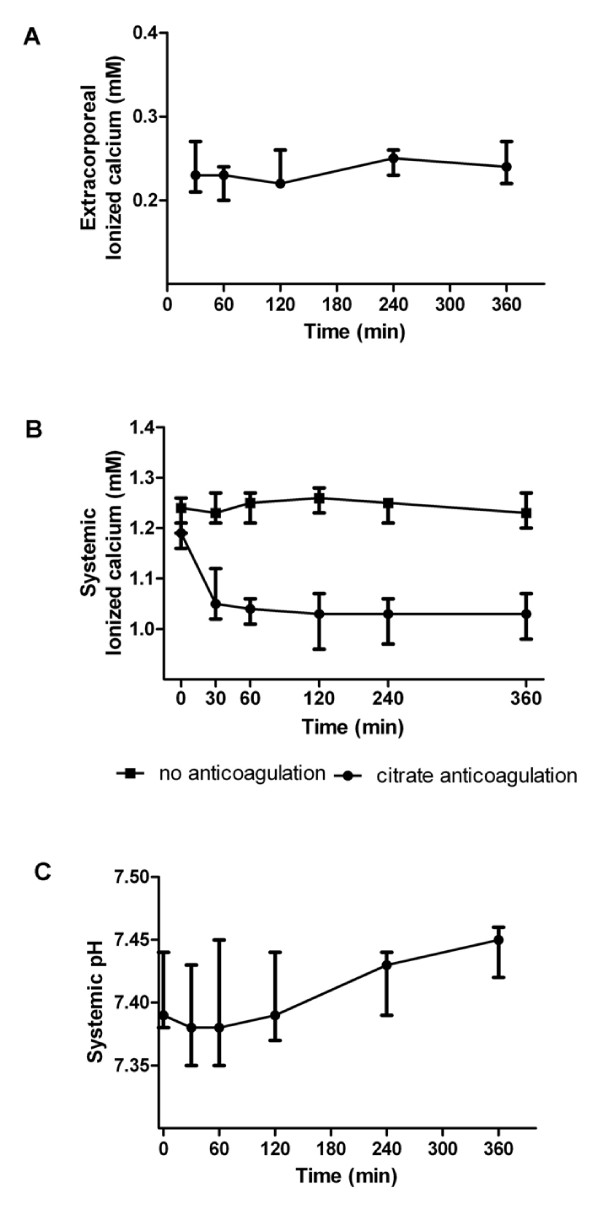
**Parameters of citrate anticoagulation**. (**a**) Extracorporeal ionized calcium concentrations (mmol·l^-1^) at the MARS dialyzer inlet during regional citrate anticoagulation. (**b**) Systemic ionized calcium concentrations (mmol·l^-1^) in patients with regional citrate anticoagulation (closed circles) and in patients receiving MARS treatment without additional anticoagulation (closed cubes). (**c**) Systemic pH in patients treated with regional citrate anticoagulation. MARS, Molecular Adsorbents Recirculation System.

We monitored adequacy and safety of regional citrate anticoagulation during MARS treatment in patients with acute-on-chronic liver failure. Ionized calcium concentration targets (0.2 ≤ Ca^2+ ^≤ 0.3 mmol·l^-1^) at the MARS dialyzer inlet were reached in all patients (Figure 3a). Systemic ionized calcium concentrations (Figure 3b) were significantly reduced during citrate anticoagulation as compared to anticoagulation-free MARS sessions (*P *< 0.001, at 30, 60, 120, 240 and 360 min). In two patients, systemic ionized calcium concentrations dropped below 0.8 mmol·l^-1^, triggering per protocol bolus infusions of CaCl_2_. Overall, reductions in systemic ionized calcium concentrations were modest and did not result in clinical symptoms. Overall, ionized calcium concentrations remained in the physiological range in all patients at all time points.

In addition, we monitored the ratio of total blood calcium to ionized calcium concentrations (Ca_tot_/Ca^2+^). As Ca_tot _represents the sum of ionized, protein-bound and citrate-bound calcium, increased ratios are considered a marker of citrate accumulation. During three sessions, we observed a ratio slightly exceeding 2.5. It is important to notice that ratios returned to baseline at the start of the next MARS session in all patients. Acid-base status was similar at all individual time points and did not significantly differ from sessions without heparin, excluding massive accumulation of citrate.

## Discussion

We performed a prospective randomized controlled trial on anticoagulation protocols during Molecular Adsorption Recirculating System (MARS) treatment. Regional citrate anticoagulation of the extracorporeal circuit under strict metabolic monitoring appears safe, feasible and proves superior to MARS treatment without anticoagulation.

Liver failure is a heterogeneous disease state resulting from either acute or chronic relentless loss of liver synthetic or detoxification function. To improve outcomes of this grave condition, several experimental (bio-)artificial devices have been developed, including fractionated plasma separation and adsorption (FPSA) and the molecular adsorption and recirculating system (MARS) [2]. Patency of the extracorporeal blood circuit is a prerequisite for all of these therapies.

Anticoagulation using unfractionated heparin given to maintain patency of the extracorporeal device exerts systemic effects. This may tipple over a balanced but brittle coagulation system such as seen in liver failure [3] towards severe bleeding. On the contrary, insufficient anticoagulation leads to contact activation during passage of the extracorporeal circuit, thereby inducing clotting of the circuit and/or increasing the risk of thrombosis after coagulation propagation.

Regional citrate anticoagulation might prove a valid alternative. Citrate efficiently reduces extracorporeal circuit ionized calcium concentration, a necessary cofactor for coagulation. Recalcification normalizes anticoagulation prior to returning blood to the systemic circulation. In hemodialysis patients, regional citrate anticoagulation proves a valid alternative for unfractionated heparin and low molecular weight heparins [11]. The downside of regional citrate anticoagulation is that only part of the used citrate is eliminated by the extracorporeal device, whereas the other part is returned to the patient, the amount of which depends on the extracorporeal device. Citrate is metabolized by the liver and to a lesser extent by other tissues, for example, the intestines and the kidneys. While this appears safe in patients with end-stage kidney disease, it has long been held that liver dysfunction and especially liver failure is a contra-indication for use of regional citrate anticoagulation. Recently, several observational studies suggest that citrate anticoagulation can be safely used in patients with liver failure treated with either FPSA or MARS [14,15]. In this study, we confirm that regional citrate anticoagulation for MARS is safe and feasible.

A critical aspect not touched upon previously is a comparison between these anticoagulation strategies. This is the first prospective randomized study to demonstrate superior patency during MARS using regional citrate anticoagulation when compared to no anticoagulation. The primary endpoint was longevity of prescribed MARS treatment sessions. Three out of 13 MARS sessions performed without anticoagulation were interrupted preterm due to occlusive clotting of the extracorporeal circuit, in line with previous observations (Table 1) [10]. In contrast, none of the treatment sessions using regional citrate anticoagulation ended preterm (Log rank *P *= 0.03).

This translates in significantly higher RRs of total bilirubin (*P *= 0.02) in patients when citrate anticoagulation was used. When analyzing completed treatment sessions only, RRs were similar. This suggests that observed differences should predominantly be ascribed to treatment downtime rather than to reduced permeability of clotted dialysis fibers.

The current study has several limitations. Due to the cross-over design all patients are treated with both citrate anticoagulation and without anticoagulation. This precludes between groups analysis of longer-term outcomes. Second, the number of patients is small, increasing the risk of type I error. Finally, liver failure is a heterogeneous disease state. For all of these reasons we opted for a cross-over design, where each patient is his/her own control, thereby minimizing the effects of heterogeneity and increasing the statistical power. Nevertheless, while regional citrate anticoagulation provides superior patency and increased treatment efficacy as compared to no anticoagulation, the available metabolic data need confirmation in a larger cohort of patients. We do not have data on systemic citrate accumulation. Observed ratios of total calcium over ionized calcium ruled out important accumulation of citrate, thus reassuring safety of regional citrate anticoagulation during MARS treatment. Nevertheless, continuous vigilance when using citrate anticoagulation in patients with liver failure is warranted, as these patients have a diminished citrate clearance. An observation worth noticing is that measurement of ionized calcium concentration might be biased due to access recirculation. We, therefore, recommend the use of a separate arterial line which, given the setting of the patients, most often is in place.

## Conclusions

Avoidance of anticoagulation during MARS results in significant loss of treatment efficacy due to treatment downtime. Regional citrate anticoagulation for MARS treatment is feasible. The metabolic tolerance is good, but continuous vigilance and strict metabolic monitoring remains warranted. Additional studies are required to identify the optimal anticoagulation regimen for extracorporeal circulation in patients with liver failure.

## Key messages

• Regional citrate anticoagulation under strict metabolic monitoring is feasible in patients with liver failure treated with the MARS system.

• Avoidance of anticoagulation during MARS results in significant loss of treatment efficacy due to treatment downtime.

• Additional studies are required to identify the optimal anticoagulation regimen for extracorporeal circulation in patients with liver failure.

## Abbreviations

aPTT: activated partial thromboplastin time; *C*_post_: concentration at the end of treatment; *C*_pre: _concentration at the start of treatment; CVVH: continuous veno-venous hemofiltration; FPSA: Fractionated Plasma Separation and Adsorption system; MARS: Molecular Adsorbent Recycling System; PT: prothrombin time; RR: reduction ratio; SD: standard deviation; TAT: thrombin-antithrombin complex.

## Competing interests

The authors declare that they have no competing interests.

## Authors' contributions

BM conceived of the study, participated in its design and coordination, performed sample collection and helped to draft the manuscript. FN participated in study design. PV performed biochemical analyses and helped to draft the manuscript. WL, AW and PE participated in study design and coordination and helped to draft the manuscript. All authors read and approved the final manuscript.
